# Are Human Mating Preferences with Respect to Height Reflected in Actual Pairings?

**DOI:** 10.1371/journal.pone.0054186

**Published:** 2013-01-16

**Authors:** Gert Stulp, Abraham P. Buunk, Thomas V. Pollet, Daniel Nettle, Simon Verhulst

**Affiliations:** 1 Department of Psychology, University of Groningen, Groningen, The Netherlands; 2 Department of Behavioural Biology, University of Groningen, Groningen, The Netherlands; 3 Royal Netherlands Academy of Arts and Sciences, Amsterdam, The Netherlands; 4 Department of Social and Organizational Psychology, VU University Amsterdam, Amsterdam, The Netherlands; 5 Centre for Behaviour and Evolution, Institute of Neuroscience, Newcastle University, Newcastle, United Kingdom; University of Turku, Finland

## Abstract

Pair formation, acquiring a mate to form a reproductive unit, is a complex process. Mating preferences are a step in this process. However, due to constraining factors such as availability of mates, rival competition, and mutual mate choice, preferred characteristics may not be realised in the actual partner. People value height in their partner and we investigated to what extent preferences for height are realised in actual couples. We used data from the Millennium Cohort Study (UK) and compared the distribution of height difference in actual couples to simulations of random mating to test how established mate preferences map on to actual mating patterns. In line with mate preferences, we found evidence for: (i) assortative mating (*r* = .18), (ii) the male-taller norm, and, for the first time, (iii) for the male-not-too-tall norm. Couples where the male partner was shorter, or over 25 cm taller than the female partner, occurred at lower frequency in actual couples than expected by chance, but the magnitude of these effects was modest. We also investigated another preference rule, namely that short women (and tall men) prefer large height differences with their partner, whereas tall women (and short men) prefer small height differences. These patterns were also observed in our population, although the strengths of these associations were weaker than previously reported strength of preferences. We conclude that while preferences for partner height generally translate into actual pairing, they do so only modestly.

## Introduction

Finding a mate to form a reproductive unit is a complex process but an important factor in determining an individual’s Darwinian fitness. Mating preferences, the propensity to mate with certain phenotypes [1], are an important part of pair formation. However, due to constraints in the mating process the preferred partner characteristics may differ from actual partner characteristics when a pair is formed. For instance, limited availability of mates and hence severe competition with rivals may prevent one from ending up with the desired partner [2], [3]. In addition to such constraints, the risk of being deserted for a better option after pair formation may make it strategically optimal to forego mating options with members of the opposite sex that are preferred by many, to ensure a long-term pair bond [4]. This consideration arises because even when a pair is formed, the availability of attractive alternatives is a determinant of the stability of that pair [5], [6].

In addition, many characteristics are taken into account when choosing a mate [7], which likely results in choosing a mate with some preferred, but other less-preferred characteristics, even when choice is without constraints. A mismatch between actual and preferred mate characteristics is even more pronounced when a desired characteristic is traded off against another one, implying that selecting on one desired characteristic reduces the likelihood of obtaining a different preferred characteristic (as suggested for example for parental investment and genetic quality; [8], [9]). An additional obstacle for obtaining a preferred partner arises when there is mutual mate choice, in which case the preferences and choice of the opposite sex further complicate the mating process [10]. All of the above reasons may lead to pair formation where both individuals have a less than ideally preferred partner.

It seems likely that the translation of preferences into actual partner characteristics will be constrained, causing a mismatch between preferences and actual mating patterns, yet this mismatch has been little studied. Here we test whether preference rules with respect to human height are translated in actual pairings. Human height is a partner characteristic that is valued by both men and women and preferences for partner height have been well studied (reviewed in [11]). These preferences can be described as the following set of rules: assortative mating, the male-taller norm, the male-not-too-tall norm, and preferences for partner height differences are dependent on one’s height. Although the above preferences have consistently been shown in Western populations using a variety of methodologies, partner height preferences and choice may be different in non-Western populations ([12]–[15]; see [16] for potential causes for these differences). In this paper, we focus exclusively on Western mating preferences for height, and below we describe these in more detail before going on to test whether these preference rules are translated in actual pairings.

### Assortative Mating

In both men and women, questionnaire based data suggest that with increasing height the preferred partner height also increases [11], indicating preferences for assortative mating. Similar patterns have been found in responses to online advertisements [17] and in speed dating events [18]. Assortative preferences for height seem to be realised in actual couples [19]–[23]. Spuhler (1982), for instance, reviewed assortative mating with respect to physical height in 28 populations and found an average between partner height correlation of.2 [23].

### Male-taller Norm (Female-shorter Norm)

In general, women prefer men taller than themselves and, conversely, men prefer women shorter than themselves [11], [24]–[26]. Again, preferences are reflected in pairings as the male-taller norm is also found in actual couples. Gillis and Avis (1980) found that in only 1 out of 720 US/UK couples, the female was taller [19]. Because women are on average shorter than men, chance predicts that the occurrence of couples in which the female is taller is 2 out of 100, 14 times higher than the observed 1 out of 720 (see [12] for a recent study replicating this finding in a Western population).

### Male-not-too-tall Norm (Female-not-too-short Norm)

Not only do men and women prefer the male to be taller than the woman in a romantic couple, they also prefer the male not to be too tall relative to the woman: the male-not-too-tall norm [11], [24]–[26]. In a sample of undergraduates selecting dates, the largest reported acceptable height difference for both sexes was the male being 17% taller than the female [25]. The extent to which the male-not-too-tall norm is expressed in actual couples is currently unknown, and in the present study we address this issue.

### Preferred Partner Height Differences are Dependent on One’s Own Height

According to Pawlowski (2003), preferred partner height difference depends on an individual’s own height [24]: both shorter men and taller women prefer smaller partner height differences than taller men and shorter women do, who prefer larger partner height differences. However, it is not known whether these preferences for partner height differences are realised in actual couples, and we therefore also address this issue.

To test to what extent the above described rules with respect to preferences for partner height are realised in actual couples, we compared the distribution of actual couple heights to the distribution of couple heights expected when mating was random with respect to height. With this technique, we were able to statistically assess simultaneously the male-taller norm, the male-not-too-tall norm, and whether preferred partner height differences are dependent on one’s own height. We compare our estimates to those previously reported on partner height preferences, to assess how well preferences translate into pair formation [11]. Although assortative mating, the male-taller norm, and the male-not-too-tall norm may be considered as distinct preference rules, this need not be the case. For instance, strict adherence of individuals to assortative mating would lead to a male-taller and male-not-too tall norm on the population level. Through simulation techniques, we examined how enforcing either a male-taller norm, or a male-not-too-tall norm would affect the strength of assortative mating.

## Materials and Methods

### Ethics Statement

We used data from the Millennium Cohort Study (MCS). The MCS was approved by the South West and London Multi Centre Research Ethics Committees. All participants provided their written informed consent to provide their data on the understanding that this would be subsequently used in secondary analyses. The present analyses did not require additional ethics approval.

### Sample

We used data from the Millennium Cohort Study (MCS), a survey that gathered information from the parents of 18,819 babies born in the United Kingdom in 2000 (see [27]–[29] for a detailed description). In brief: parents were interviewed when their babies were 9 months old. The sample was selected from a random sample of electoral wards, disproportionately stratified to ensure adequate representation of all four regions of the UK, areas with higher minority ethnic populations, and deprived areas [27], [28], [30]. The overall response rate was 68% [30]. Height of the mother and father were self-reported. Self-reported measures of height have been shown to be very reliable (*r* >.90) [31], [32]. Nonetheless, these studies also indicate that both men and women are likely to overestimate their height; men about 1.2 cm and women about 0.60 cm [31]–[33]. These biases are unlikely to affect our conclusions. First, the bias is less pronounced below the age of fifty [31]–[33], as are the men and women in our sample [29]. Second, adding a constant value to the heights of men will not affect the correlational measures nor the results from the simulations presented. It may, however, be the case that the observed number of pairs in which the male is taller than the female (*N* = 11,566) is a slight overestimation of the actual number of pairs in which this is the case. For the analyses presented here, we included all heterosexual parents for which both heights were available (12,502 cases). Women were on average 163.75±6.97 (mean ± standard deviation) and men 177.86±7.42 centimetres tall. The average Parental Height Difference was 14.11±9.25 centimetres. Because height is related to ethnicity, and there is strong assortative mating for ethnicity we re-analyzed our data restricting our sample to Caucasian parents (*N* = 10,664). This led to very similar results (results not reported).

### Analysis

We investigated whether and how the observed distribution of Parental Height Differences (PHD; male height minus female height in cm) differed from the distribution expected under random mating over height. To obtain an estimate of PHD under random mating, we generated 10,000 samples in R [34], each sample being a complete randomization of the 12,502 couples (and thus their heights). We compared the distribution of PHD resulting from these random samples to the PHD distribution in the original population, to examine the differences between the observed heights and the heights in random mating. In order to do so, we divided the range of PHD in the original population and the 10,000 random samples in 5 centimetre bins, and counted the occurrences of these bins in both the original population and the random samples (bins with fewer than 75 cases were collapsed resulting in a lower bound cut-off bin of <-15 cm and a higher bound cut-off bin of >35 cm). For instance, the bin 15 to 20 cm, indicating that the male partner was 15 to 20 cm taller than the female, occurred exactly 2,586 times in the original population. The median value (50^th^ percentile) of occurrences of this bin in the 10,000 random samples was 2,464. This indicates that the most likely number of occurrences (median of 10,000 samples) of the bin 15–20 cm is 2,465 when mating with respect to height is random, which suggests that this bin occurred more often in the original population than expected under random mating. Ninety-five per cent of the occurrences of this bin in the 10,000 samples fell between 2,382 (the 2.5^th^ percentile) and 2,549 (the 97.5^th^ percentile). The actual value (2,586) falls outside this range, indicating that this specific bin occurred significantly more often in the original population compared to what would happen when mating was random with respect to height.

A specific *p*-value for the difference between the original and the random samples was determined by what proportion of the 10,000 samples the occurrence of the bins were higher, equal or lower than the actual occurrences of these bins. For instance, the bin 15 to 20 cm was found to be equally or less frequent than 2,586 (the number of occurrences of this bin in the original sample) in only 21 of the 10,000 samples. Thus, the occurrence of this bin is significantly different from random mating with a *p*-value of 21/10,000 is 0.0021. This *p*-value concerns the directional hypothesis that the height bin is *either* over- or underrepresented compared to the original sample, not the hypothesis that the height bin has a different frequency in the random samples compared to the original sample, and as such is one tailed.

For every PHD bin, we also calculated the ‘relative likelihood of pairing’, the frequency of observing a particular PHD bin in the original population relative to random mating, by dividing the number of occurrences in the actual population of that PHD bin by the median number of occurrences of that PHD bin in the random samples. For example, the frequency of the PHD bin 15 to 20 cm was 2,586 in the actual original population, which we divided by 2,464 (median occurrence in 10,000 samples of random mating), yielding and 1.05 implying this PHD bin is 5% more frequent than expected by chance. A relative likelihood of pairing greater (*lower*) than one means that the PHD bin is more (*less*) likely to occur in the actual population than expected by random mating.

## Results

### Assortative Mating

**Figure 1:**
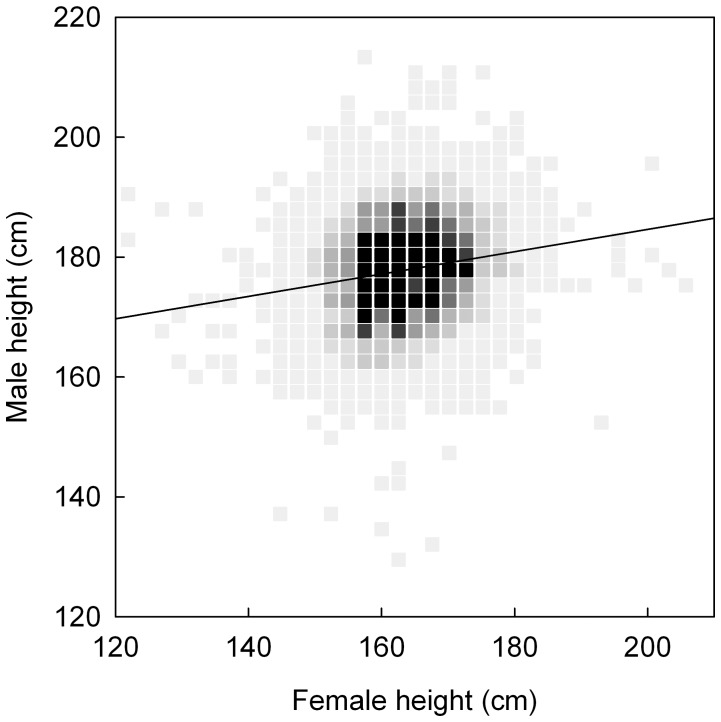
The positive correlation between female and male height (*r = *.18). Lumination indicates frequency of occurrence (lightest color <20 couples; darkest color >200 couples).

We first examined whether assortative mating over height, the male-taller norm, and the male-not-too-tall norm were apparent in our sample. In line with earlier studies [19]–[23], we found that taller women had taller partners, indicating assortative mating with respect to height (*r* = .18; *p*<.0001; Figure 1). For every cm increase in female height, partner height on average increased with 0.19 cm (i.e. the slope of the regression line; linear regression: B (± SE) = 0.19±0.01; *p*<.0001; intercept (± SE) = 147.34±1.54; *p*<.0001). Similarly, for every cm increase in male height, the female partner is predicted to increase with 0.17 cm (linear regression: B (± SE) = 0.17±0.01; *p*<.0001; intercept (± SE) = 134.47±1.47; *p*<.0001). Courtiol and colleagues [11] provide estimates for their assortative preference functions (i.e. the slope of the preference function), and find that, for women, an increase of 0.77 cm per cm (95% *CI* = 0.51 to 1.03) own height is preferred, whereas for men an increase of 0.60 cm per cm (95% *CI* = 0.37 to 0.84) own height is preferred. Thus, while taking into account that the estimates for the preference functions were taken from a different populations with potential differences in average heights and variation in height, the slopes of the preference functions are substantially and significantly larger in magnitude than the slopes of assortative mating in our sample. This suggests that the assortative preference for height is only weakly realized in actual couples.

### The Male-taller Norm

**Figure 2:**
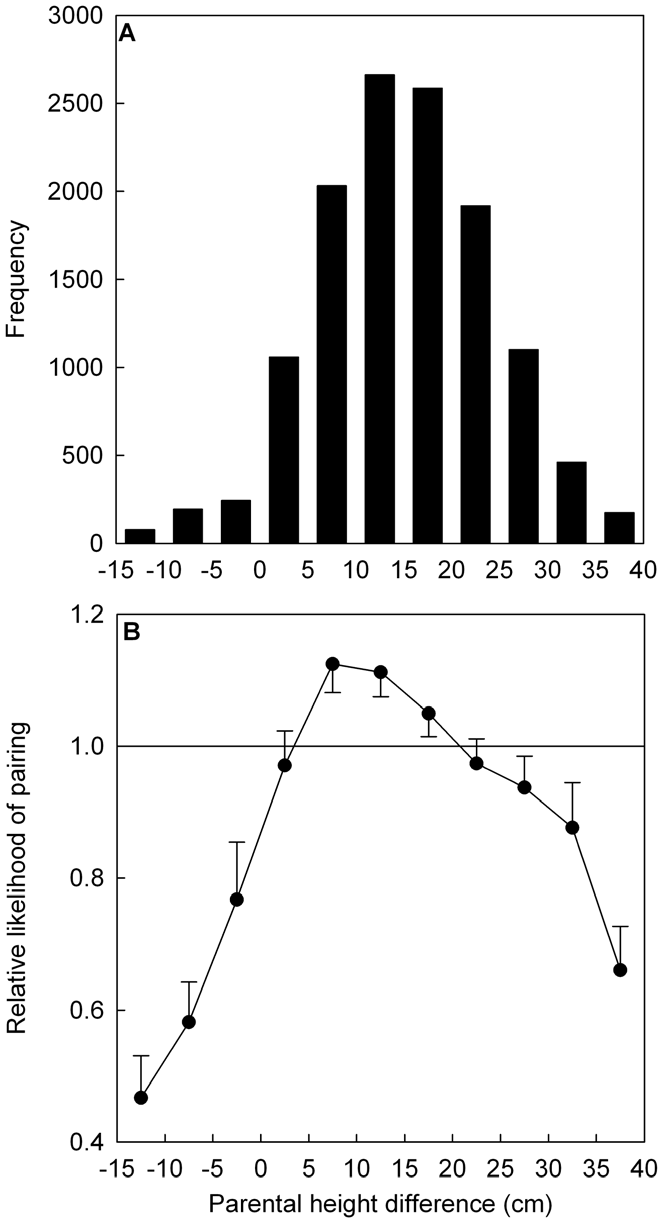
The frequency distribution of parental height differences (a) and the relative likelihood of pairing (b). Parental Height Differences (PHD) in bins of 5 cm. The relative likelihood of pairing in these bins is the frequency of the bins in the original population divided by the median (±97.5% upper/lower limit) occurrences of that bin in the 10,000 samples of random mating (see text). A number greater (*lower*) than one (solid horizontal line) means that the PHD bin is more (*less*) likely to occur in the original population than expected by random mating.

Comparing the actual occurrences of the PHD bins in the population (Figure 2A) to the expectation under random mating provided clear evidence for the male-taller norm being reflected in actual pairings (Table 1; Figure 2B). Adherence to the male-taller norm was evident in these data since men were taller than their partners in 92.5% of the couples, significantly more often than the expected 89.8% when mating was random with respect to height (*p*<.0001; Table 1). The male-taller norm was thus violated in 10.2% of the couples when mating was random, while in the original population this norm was violated in 7.5% of the couples, a 26% reduction (Table 1). Furthermore, bins in which the female was substantially taller than the male (PHD<–5 cm) were much less likely to occur compared to random mating than bins in which the females was only slightly taller than the male (Table 1; Figure 2B), indicating that when the male-taller norm was violated it was most likely violated only slightly.

**Table 1:** Occurrences of similar height partners (♂ = ♀), male taller (♂>♀) and male shorter (♂<♀) compared to female, and Parental Height Differences (PHD; male height – female height) in bins of 5 centimetre in couples from the Millennium Cohort Study (MCS) and in the 10,000 samples of random mating.

	Number of observed cases				
	MCS	10,000 Random samples			
		median	95% data range	Difference^a^	Rel. likel. pairing^b^
♂<♀	511	811	772–851	<.0001	0.63
♂ = ♀	425	460	420–499	.0442	0.92
♂>♀	11566	11231	11185–11277	<.0001	1.03
PHD (in cm)					
< −10	78	167	147–189	<.0001	0.47
−10 to −5	192	330	299–362	<.0001	0.58
−5 to −0	241	314	282–348	<.0001	0.78
0 to 5	1058	1090	1034–1146	.1372	0.97
5 to 10	2032	1807	1736–1880	<.0001	1.12
10 to 15	2663	2395	2314–2478	<.0001	1.11
15 to 20	2586	2464	2382–2549	.0021	1.05
20 to 25	1917	1969	1896–2044	.0820	0.97
25 to 30	1101	1175	1118–1232	.0056	0.94
30 to 35	461	527	488–567	.0002	0.88
>35	173	262	238–287	.0001	0.66

a *p*-value for difference of occurrence of bin between original sample and 10,000 samples of random mating sample (see text).

b The Relative likelihood of pairing is the number of occurrences of a bin (second column) divided by the median occurrences of this bin (third column) in the random samples.

### The Male-not-too-tall Norm

The male-not-too-tall norm was also reflected in the actual pairings: bins in which the male was 25 or more cm taller than their partner occurred significantly less often in the original population (13.9%) than expected when mating was random with respect to height (15.7%; Table 1; Figure 2B). Thus, 15.7% of the couples were predicted to violate the male-not-too-tall norm when mating was random (with the assumption that the norm lies at a PHD of 25 cm), while in the original population this norm was violated in 13.9% of the couples, a reduction of 12%. The intermediate range of PHD, in which the male was 5 to 20 cm taller than their female partner, occurred more often in the original population compared to random mating (Table 1; Figure 2B). Similar to the male-taller norm, we found that when the male-not-too-tall norm was violated, it was most likely violated only slightly (Table 1; Figure 2B). Thus, a height difference of 25–30 cm was relatively more likely to occur than a height difference of 30–35 cm, but both were observed less often than expected by chance (Table 1; Figure 2B).

In conclusion, in line with reported partner height preferences [11], we found evidence for assortative mating, the male-taller norm and the male-not-too-tall norm. However, the level of assortative mating (*r* = .18) is moderate, and the male-taller norm was violated in only 26% fewer pairs than expected by chance. Similarly, in 13.9% of the couples, the male-not-too-tall norm (i.e. >25 cm height difference) was violated, only 12% less than expected by chance. Thus, these preference rules are only weakly translated into actual couple formation.

### Preferred Partner Height Differences are Dependent on One’s Own Height

On the basis of reported preferences for partner height differences [24], we predicted that, when preferences are translated into actual mating patterns, taller compared to shorter men would have large partner height differences (i.e. the man being much taller than the woman). Similarly, we predicted that taller women compared to shorter women would have smaller partner height differences (i.e. the man being only slightly taller than the woman). We indeed found that taller men had greater parental height differences than shorter men, as indicated by a positive correlation between male height and PHD (*r* = .67). Similarly, we found that shorter women had greater parental height differences than taller women (*r* = –.61). However, this pattern is also observed when we randomly pair individuals. In 10,000 simulations of random pairing we find a median correlation of *r* = .73 (95% *CI* = .72 to.74) for the relationship between male height and PHD and a median of *r* = −.68 (95% *CI* = −.69 to −.68) for this relationship in women. Thus, purely random mating with respect to height generates a pattern in which taller men (and shorter women) have larger height differences than shorter men (and taller women).

To assess how well this preference rule is realized in actual couples, we again compared the estimates of our slopes from the relationship between own height and PHD to those reported in [11]. For every cm increase in female height, we showed that partner height on average increased with 0.19 cm (see above), which equals to a decrease of 0.81 cm in partner height differences. Similarly, for every cm increase in male height, we showed that partner height on average increased with 0.17 cm (see above), which equals to an increase of 0.84 cm in partner height differences. In contrast, the slopes for the preference function with respect to partner height differences for women is −0.23 cm per cm (95% *CI* = −0.49 to 0.03) own height, and for men 0.4 cm (95% *CI* = 0.16 to 0.63) [11]. Thus, the slopes from the preference function for females (−0.23) and males (0.4) were substantially smaller in magnitude compared to the slopes observed in the couples (−0.81 and 0.84 respectively). For women, on the one hand, we found that with increasing height the parental height differences *decreased more* than actually preferred. For men, on the other hand, we found that with increasing height the parental height differences *increased more* than preferred. In conclusion, and again taking into account that we have used estimates from a preference function of a different population, which can differ in both slope and intercept of the preference function from our population, we found that realized partner height differences are in line with preferences for partner height differences, although the difference in slopes suggest that the realized height differences are different from ideally preferred.

### Non-mutual Exclusive Rules

Although we have treated assortative mating, the male-taller norm and the male-not-too-tall norm as distinct rules, they are not completely independent. For example, strict assortative mating (for instance: ‘*always select a partner with a PHD that conforms to the average height difference between the sexes*’) would lead to strong adherence to both the male-taller and the male-not-too tall norm. Likewise, adhering to the male-taller norm will by itself generate assortative mating with respect to height. To examine the relationships between these norms on the one hand, and assortative mating on the other hand, we randomly coupled partners in 10,000 generated samples, while forcing either a male-taller norm (*‘as a female accept any partner taller than you’*) or a male-not-too-tall norm (*‘as a female accept any partner that is less than 25 cm taller than you’*). We chose a value of 25 cm, because all bins above this value were significantly underrepresented in our population (Figure 2B). Because of the sequential nature of pairing in our algorithm, women that ‘chose’ last may not be able to find a partner that conforms to the norm, leaving them single. In the two times 10,000 samples (one for each norm), the percentage of unpaired individuals we observed ranged from 0 to 0.1%, which we considered low enough to ignore and we therefore excluded the unpaired individuals from our analyses. When forcing a male-taller norm, we observed a median correlation between partner heights of *r* = .34 (95% CI:.33–.35), which was almost twice as high as the correlation of assortative mating in the population (*r* = .18). When a male-not-too-tall norm was enforced we observed an even higher median correlation between partner heights of *r* = .47 (95% CI:.46–.48). Increasing the value of the norm (i.e. >25 cm) lowers the median correlation, whereas decreasing this value increases it (results not reported). In conclusion, adhering to either a male-taller norm or a male-not-too-tall norm results in significant positive assortment for height, much stronger than observed in the actual population. This indicates that either norm in isolation would suffice to generate the pattern of assortative mating for height found in the population.

## Discussion

Preferences with respect to specific characteristics are an important ingredient of pair-formation, but multiple constraints (see Introduction) may prevent the realisation of such preferences when forming a pair. In this study, using simulations in which we randomized pairings, we examined whether previously documented preference rules for partner height were realised in actual couples. Firstly, we replicated the well-known finding that there is assortative mating with respect to height (Figure 1). We also replicated the finding of a male-taller norm (Figure 2), as men were more frequently taller than their partner than expected by chance. We extended this finding by showing that couples in which the man is much shorter than the woman are relatively less likely to occur than couples in which the man is only slightly shorter than the woman. Thus, when the male-taller norm is violated, it is mostly violated only slightly. A male-not-too-tall norm has previously been documented as a preference [11], [24]–[26], and we show, to our best knowledge for the first time, that this norm is translated in actual pairing (Figure 2B). Couples in which the male was more than 25 cm taller than the female partner, were rarer than expected by chance. Furthermore, similar to the male-taller norm, when the male-not-too-tall norm was violated, it was most likely violated only slightly (e.g. a partner height difference of 30 cm was relatively more likely to occur than a partner height difference of 35 cm, but both were less likely to occur than expected by chance). Lastly, in line with preferences for partner height differences, we found that shorter women and taller men were more likely to have greater partner height differences, whereas shorter men and taller women were more likely to have smaller partner height differences.

Although all known preference rules for height were qualitatively realised in actual couples, these effects were generally modest when compared to random mating. There may be several reasons for why an individual’s preferred partner characteristics differs from actual partner characteristics (see Introduction). Men and women, for instance, do not agree on their preferred partner height, as women prefer larger partner height differences than men [11]. Mutual mate choice is thus likely to produce couples in which partner height preferences for either the male, or the female, or both are not optimally satisfied. Furthermore, height is but one of many characteristics valued in a mate [35], and the strength of the preference for height in comparison to other preferred traits determines final pairing with respect to height [36]. One of the few studies examining the interplay between preferences and pairing [36], found that preferences for height, weight, and BMI were about equally strongly related to actual partner characteristics in both men and women, suggesting that these different traits are given roughly equal weight when considering a partner.

The observed non-random pairing with respect to height need not be a consequence of mating preferences with respect to height [11], [36]. It could also arise when assortment took place on a different characteristic but related to height (e.g. ethnicity and education). For instance, when there are differences in height between sub-populations, and individuals are more likely to pair within sub-populations than between sub-populations, than assortative mating for height could arise on the population level without playing a role in the pairing within sub-populations. Educational levels, for instance, may be considered as sub-populations. Height is positively related to education [37], and assortative mating for education is widely observed [21]. Thus, the correlation between partner heights might therefore at least in part be a consequence of the correlation between the educational attainments of the partners. It seems unlikely however, that these associations can fully explain the observed patterns. Firstly, the variation in height differences is much larger within a sub-population than between sub-populations (e.g. between 1–3 cm; [38]). Therefore, that height differences above 25 cm occur less often than expected by chance (i.e. the male-not-too-tall norm), is unlikely to be due to sub-population effects, because height differences between sub-populations are much smaller [38]. Secondly, assortative pairing for other characteristics than height is unlikely to result in a male-taller norm. For these two reasons we believe it is unlikely that the non-random pairing with respect to height is a consequence of assortative mating for other characteristics.

Due to the nature of our sample (i.e. parents) we excluded childless pairs, which may limit the generality of our conclusions because the proportion of childlessness is known to be related to height [39], [40]. We do, however, believe that the inclusion of childless individuals would not change our results qualitatively for two reasons. Firstly, relationships between height and measures of reproductive success are weak, typically explaining less than 1% of the variance [39]–[41]. Thus, the effect of being childless on the height distributions in our sample will be very small.

In conclusion, we have shown that all previously documented preference patterns for partner height are at least qualitatively realised in actual pairings. We note, however, that compared to random mating the magnitude of these effects was generally low, suggesting that mating preferences were only partially realised. These results are in line with a recent study that showed that traits considered strongly related to attractiveness, such as height, are not necessarily strongly related to actual pairing [36].
